# Supplementary material for: Cryo-EM structure of cell-free synthesized human histamine 2 receptor/Gs complex in nanodisc environment
Source: Nat Commun. 2024 Feb 28;15:1831. doi: 10.1038/s41467-024-46096-z (PMC10901899; doi:10.1038/s41467-024-46096-z)
Supplement: Supplementary file 1 — Supplementary Information [file 41467_2024_46096_MOESM1_ESM.pdf]

Cryo-EM structure of cell-free synthesized human histamine 2 receptor/G_s complex in nanodisc environment

Supplementary information

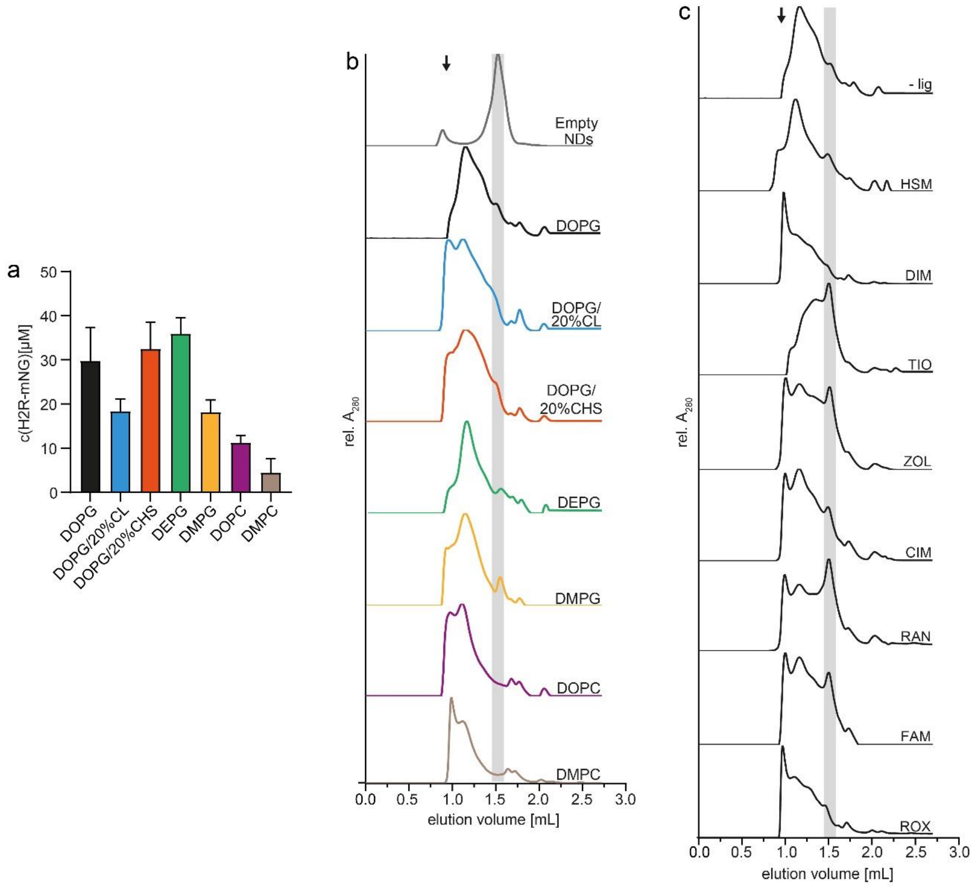

Supplementary Fig. 1. Effect of lipids and supplied ligands on H_2R solubilization and sample quality. a. Cotranslational solubilization of H_2R-mNG in different lipid environments. H_2R-mNG was synthesized in presence of 60 µM NDs pre-assembled with the indicated lipids. The H_2R-mNG/ND complexes were Strep-purified and quantified according to the mNG fluorescence. Data are presented as mean (SD) (n = 3 individual experiments). c(H_2R-mNG) [µM] means concentration of the H_2R-mNG in µM. Source data are provided as Source Data file. **b.** SEC profiles of Strep-purified H_2R/ND complexes without mNG moiety and CF synthesized in presence of 60 µM preformed NDs containing membranes with the indicated lipids. H_2R synthesis in the individual reactions corresponds to the rates given in panel a. **c.** SEC profiles of H_2R CF synthesized in presence of 60 µM NDs (DOPG) and in presence of supplied ligands. The CF synthesis rate for all reactions was comparable and approx. 30 µM final concentration. –lig: without ligand; HSM: 5 mM histamine; DIM: 5 mM dimaprit; Tio: 200µM tiotidine; ZOL: 200µM zolantidine; CIM: 500 µM cimetidine; RAN: 200 µM ranitidine; FAM: 200 µM famotidine; ROX: 200 µM roxatidine. SEC was performed with a Superose 6 3.2/300 column at a flow rate of 0.05 mL/min. Void volumes are indicated by an arrow; Peaks are normalized relative to the most prominent fraction; Fractions of proposed folded H_2R are indicated in grey.

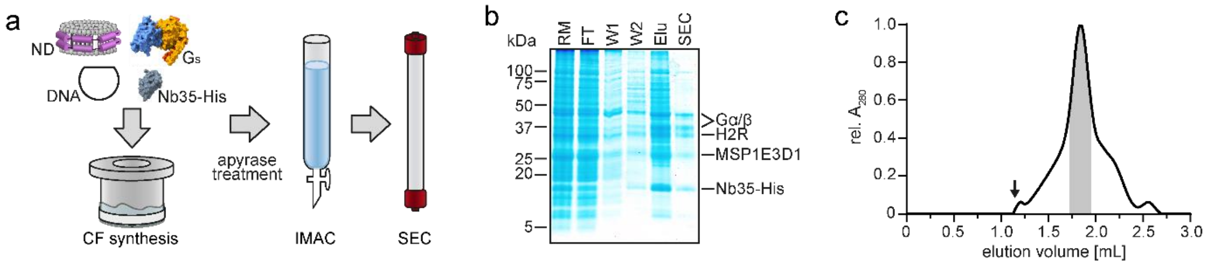

Supplementary Fig. 2. Cryo-EM sample production workflow. a. H_2R is synthesized in presence of empty, pre-formed NDs, G_s heterotrimer and Nb35-His. After apyrase treatment, the sample is subsequently purified by IMAC and SEC. **b.** SDS-PAGE of the H_2R/ND/G_s/Nb35-His complex purification process. RM: reaction mix, FT: flow through, W1: first wash, W2: second wash, Elu: elution. **c.** SEC profile of the purified H_2R/ND/G_s/Nb35-His complex. Gelfiltration was performed with a Superose 6 5/150 Increase column at a flow rate of 0.15 mL/min. The void volume is indicated by an arrow. The fractions collected and concentrated for cryo-EM analysis are indicated in grey. Fig. 2a was created by Adobe Illustrator CS6.

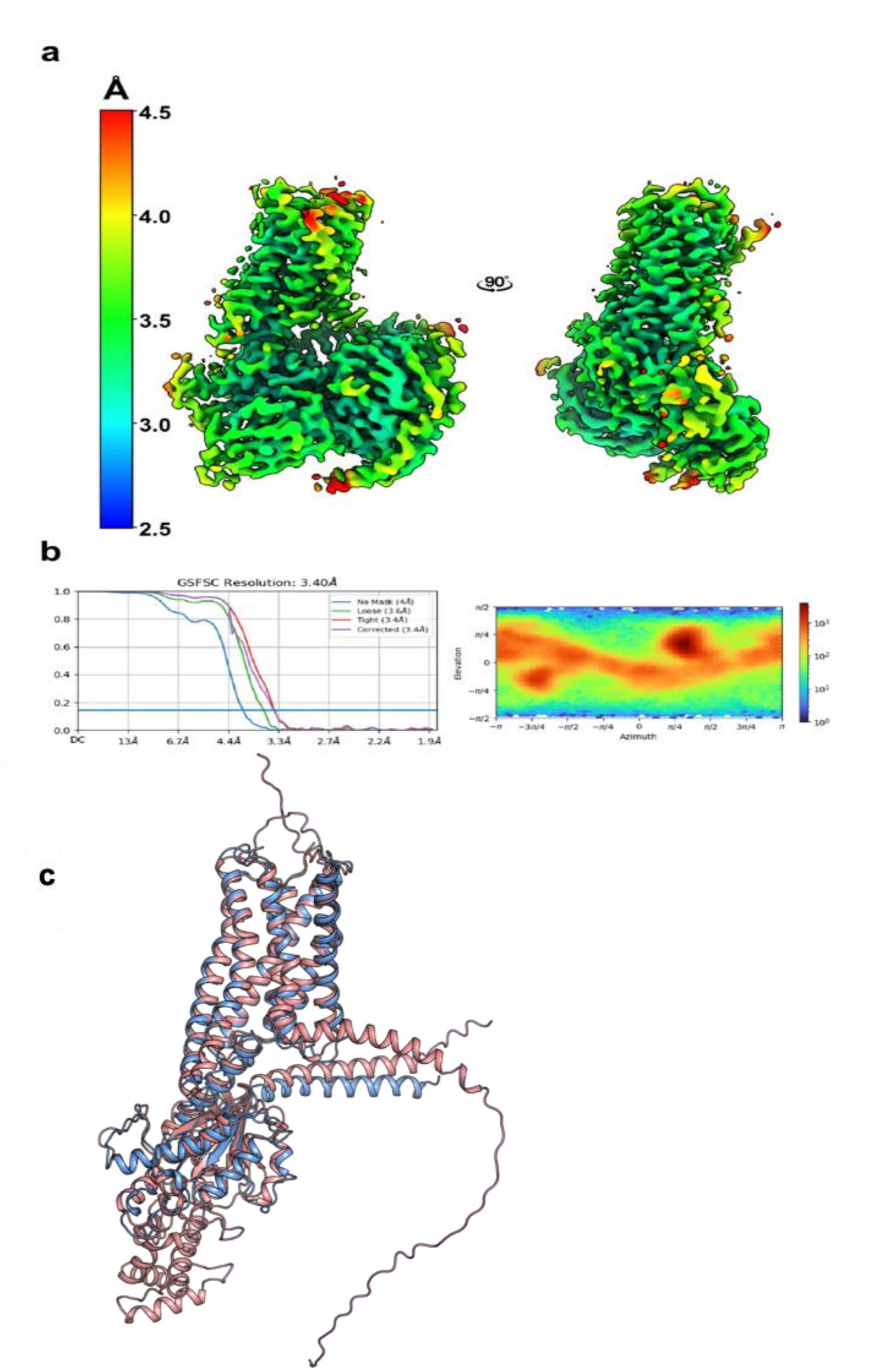

Supplementary Fig. 3. Resolution of the H_2R/G_q complex. a. Local resolution map of the H_2R/G_q complex colored according to resolution. **b.** FSC curve and particle distribution for the displayed map. **c**. Superposition of our experimental complex structure (blue) with the AF2-generated complex model (pink).

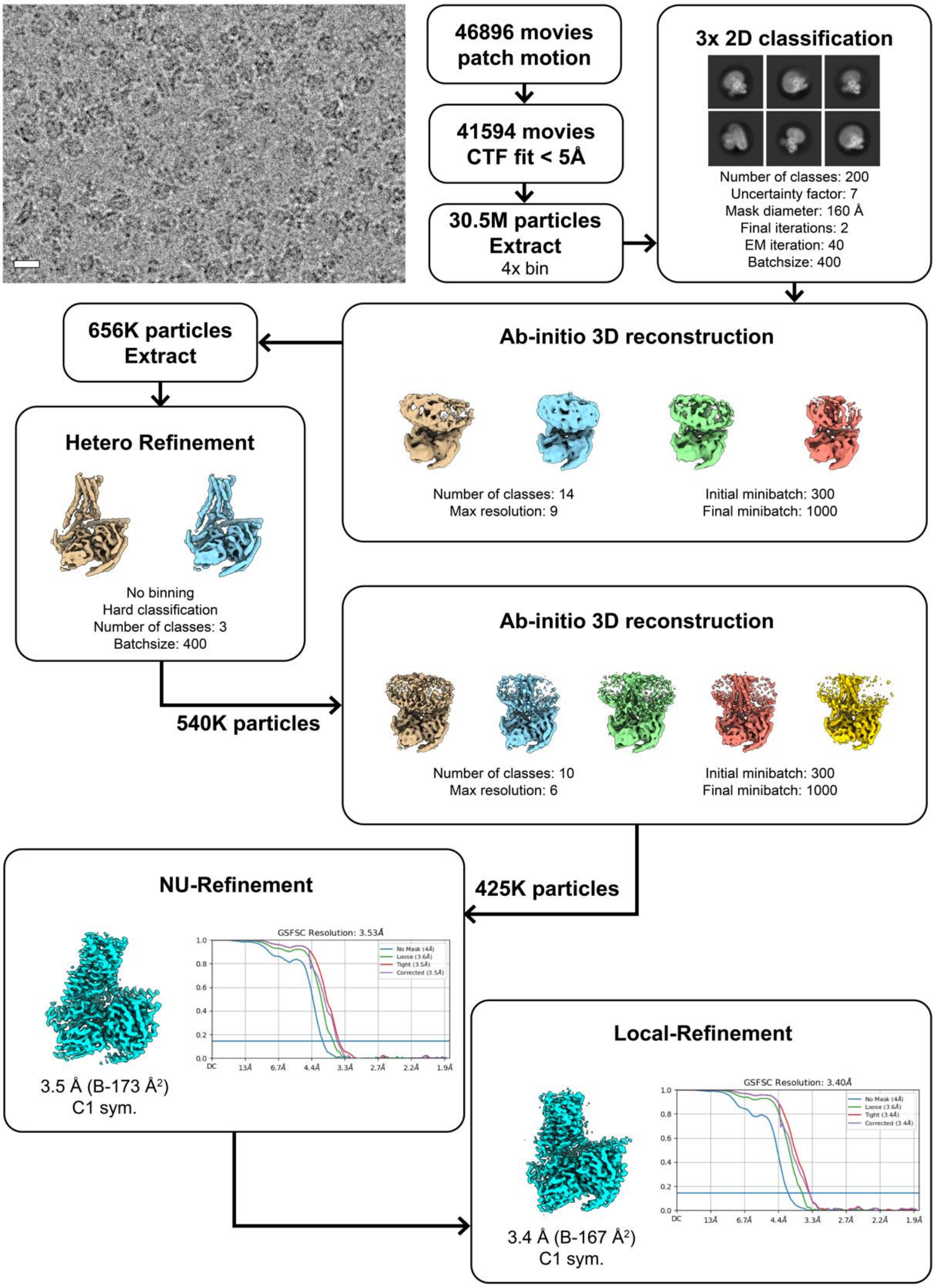

Supplementary Fig. 4. Cryo-EM data processing workflow. Processing flow chart for the H_2R/G_q complex. Processing was performed in cryoSPARC. A representative cryo-EM micrograph, along with 2D-class averages and with a 100 Å scale bar in the micrograph is shown. Only the selected classes used for further processing were displayed in *ab-initio* 3D reconstruction and hetero refinement charts.

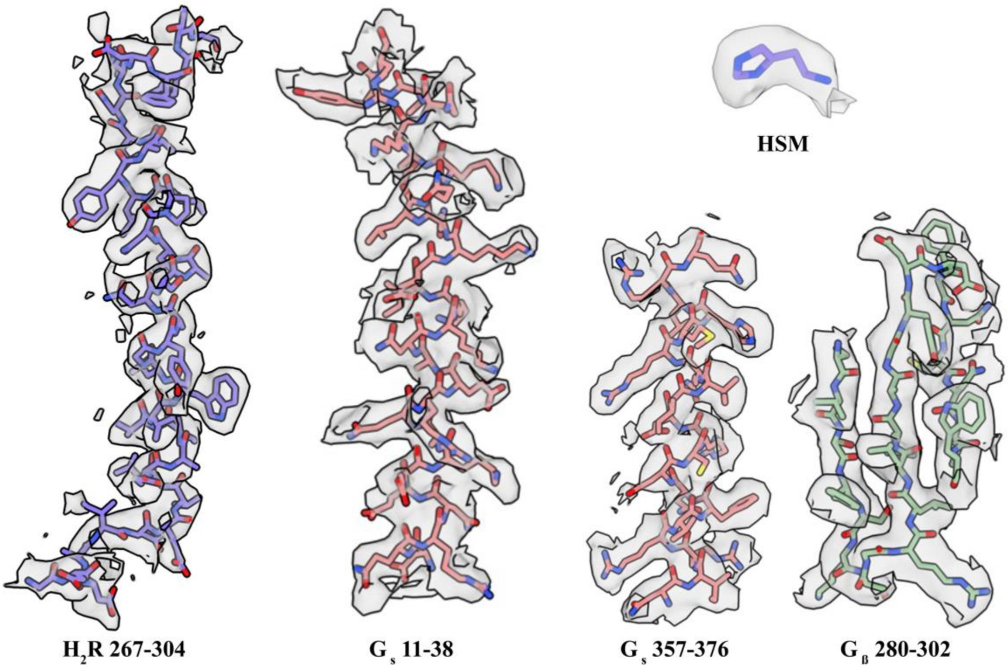

Supplementary Fig. 5. Representative cryo-EM densities of H_2R with fitted models. Shown are the densities of TM7 from amino acid position 267-304 of H_2R, of the α-helices 11-38 and 357-376 of G_s, of the β-sheet 280-302 of G_β and of the agonist histamine (HSM).

```
H2R

H2R    1  ...MAPNG..TASSFCLDSTACK..........ITITVVLAVLILITVAGNVVVCLAVG
H1R    1  ..MSLPN.....SSCLLEDKMCEGNKTTMASPQLMPLVVVLSTICLVTVGLNLLVLYAVR
H3R    1  MERAPPDGPLNASGALAGEAAAAGGARGFSAAWTAVLAALMALLIVATVLGNALVMLAFV
H4R    1  ....MPD..............TNSTINLSLSTRVTLAFFMSLVAFAIMLGNALVILAFV

H2R

H2R   45  LNRRLRNLTNCFIVSLAITDLLLGLLVLPFSAIYQLSCKWSFGKVFCNIYTSLDVMLCTA
H1R   54  SERKLHTVGNLYIVSLSVADLIVGAVVMPMNILYLLMSKWSLGRPLCLFWLSMDYVASTA
H3R   61  ADSSLRTQNNFFLLNLAISDFLVGAFCIPLYVPYVLTGRWTFGRGLCKLWLVVDYLLCTS
H4R   42  VDKNLRHRSSYFFLNLAISDFFVGVISIPLYIPHTLF.EWDFGKEICVFWLTTDYLLCTA
                                                         ★

H2R

H2R  105  SILNLFMISLDRYCAVMDPLRYPVLVTPVRVAIS......LVLIWVISITLSFLSIHL..
H1R  114  SIFSVFILCIDRYRSVQQPLRYLKYRTKTRASATILGAWFLSFLWVIPI........L..
H3R  121  SAFNIVLISYDRFLSVTRAVSYRAQQGDTRRAVRK.....MLLVWVLAFLLYGPAI.L..
H4R  101  SVYNIVLISYDRYLSVSNAVSYRTQHTGVLKIVTL.....MVAVWVLAFLVNGPMI.LVS

H2R

H2R  157  .GWNS.RNETSKGNHTTSKCKVQVNEV..YGLVDGLVTFYLPLLIMCITYYRIFKVARDQ
H1R  164  .GWNHFMQQTSV..RREDKCETDFYDVTWFKVMTAIINFYLPTLLMLWFYAKIYKAVRQH
H3R  173  .SWEYLSGGSSI..PEG.HCYAEFFYNWYFLITASTLEFFTPFLSVTFFNLSIYLNIQRR
H4R  155  ESWK.........DEGSECEPGFFSEWYILAITSFLEFVIPVILVAYFNMNIY......
                              ★

H2R

H2R  213  ..................................AKRINHISS...WK..........
H1R  221  CQHRELINRSLPSFSEIKLR........PENPKGDAKKPGKESP...WEVLKRKPKDA.
H3R  229  ................TRLRLDGAREAAGPEPPP..EAQPSPPPPPGCWGCWQKGHGEAM
H4R  199  ...............................................WSLWKRDH....

H2R

H2R       ............................................................
H1R  268  .......................GGGSVLKSPSQTPKEMKSPVVFSQEDDREVDKLYCF
H3R  271  PLHRYGVGEAAVGAEAGEATLGGGGGGGSVASPTSSSGS....SSRGTERPR........
H4R  207  .LSR...CQSHPGLTAVSSNICGHSFRGRLSSRRSLSASTEVPASFHSERQRR.......

H2R

H2R  224  .....................................AATI.................
H1R  304  PLDIVHMQAAAEGSSRDYVAVNRSHGQLKTDEQGLNTHGASEISEDQMLGDSQSFSRTDS
H3R  319  ........SLKRGSKPSAS..................SASL.EKRMKMVSQSFT....
H4R  256  ......KSSLMFSSRTKMN..................SNTI.ASKMGSFSQSDSVA..

H2R

H2R  228  .............................................REHKATVTLAAVMGA
H1R  364  DTTTETAPGKGKLRSGSNTGLDYIKFTWKRLRSHSRQYVSGLHMNRERKAAKQLGFIMAA
H3R  346  .................................QRF...RLSRDRKVAKSLAVIVSI
H4R  287  ...........................LHQREHV...ELLRARRLAKSLAILLGV

H2R

H2R  243  FIICWFPYFTAFVYRGLRGDDAIN.EVLEAIVLWLGYANSALNPILYAALNRDFRTGYQQ
H1R  424  FILCWIPYFIFFMVIAF.CKNCCN.EHLHMFTIWLGYINSTLNPLIYPLCNENFKKTFKR
H3R  367  FGLCWAPYTLLMIIRAACHGHCVP.DYWYETSFWLLWANSAVNPVLYPLCHHSFRRAFTK
H4R  312  FAVCWAPYSLFTIVLSFYSSATGPKSVWYRIAFWLQWFNSFVNPLLYPLCHKRFQKAFLK

H2R

H2R  302  LFCCRLANRNSHKTSLRSNASQLSRTQSREPRQQEEKPLKLQVWSGTEVTAPQGATDR
H1R  482  IL..........HIRS..........................................
H3R  426  LLCPQ.........KLKI...........QPHSSLEHCWK..................
H4R  372  IFC...........IKK...........QPLPS.QHSRSVSS..............
```

Supplementary Fig. 6. Amino acid sequence alignment of GPCR histamine receptors. The secondary structure symbols are illustrated according to the H_2R structure. Conserved residues are highlighted. The coordinating residues of the ligand are marked with the corresponding boxes in black. Disulfide cysteines marked with asterisks. The alignment figure was made using MAFFT multiple sequence alignment program and ESPript.

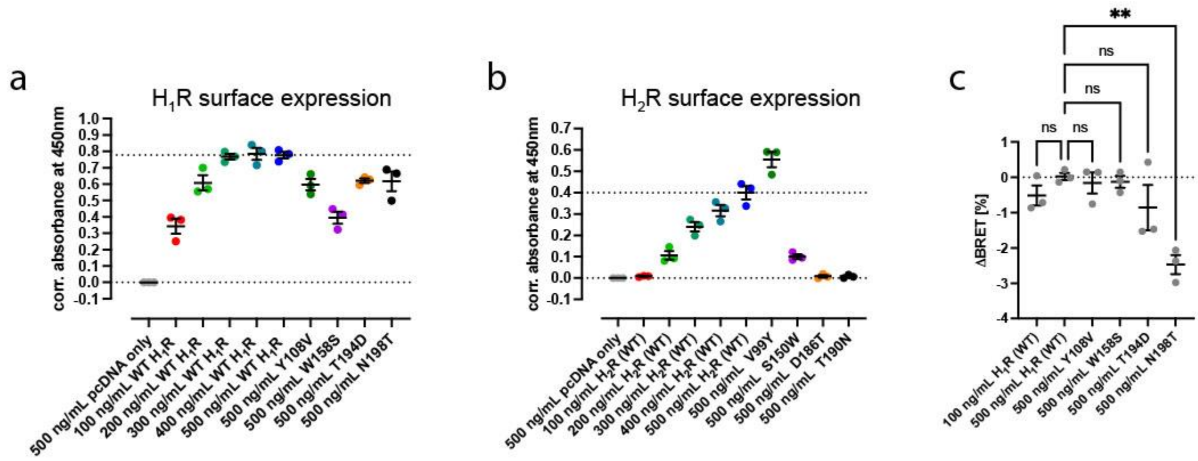

Supplementary Fig. 7. Comparative surface-expression levels of H_1R and H_2R constructs and H_1R-dependent Gq activation assay. (**a, b**) The surface-expression levels of wild-type (WT) and $H_{1/2}R$ mutants were quantified using an ELISA against the N-terminal Flag tag. Different amounts of DNA encoding for the WT receptors was used for the transfection into HEK293A cells (ng DNA/mL of transfected cells) to adjust the surface-expression level of the WT to the receptor mutants to ensure comparable protein amounts in the plasma membrane for the BRET-based G protein signaling assays. Data obtained from three independent experiments. (**c**) Comparison of the ΔBRET response of the H_1R (WT) and mutants in the presence of 100 uM amthamine showing a statistically significant decrease in the BRET signal of the H_1R-mutant N198T. Statistical significance in (c) was tested using One-Way ANOVA followed by Fisher’s LSD test for multiple comparison. Source data are provided as Source Data file.

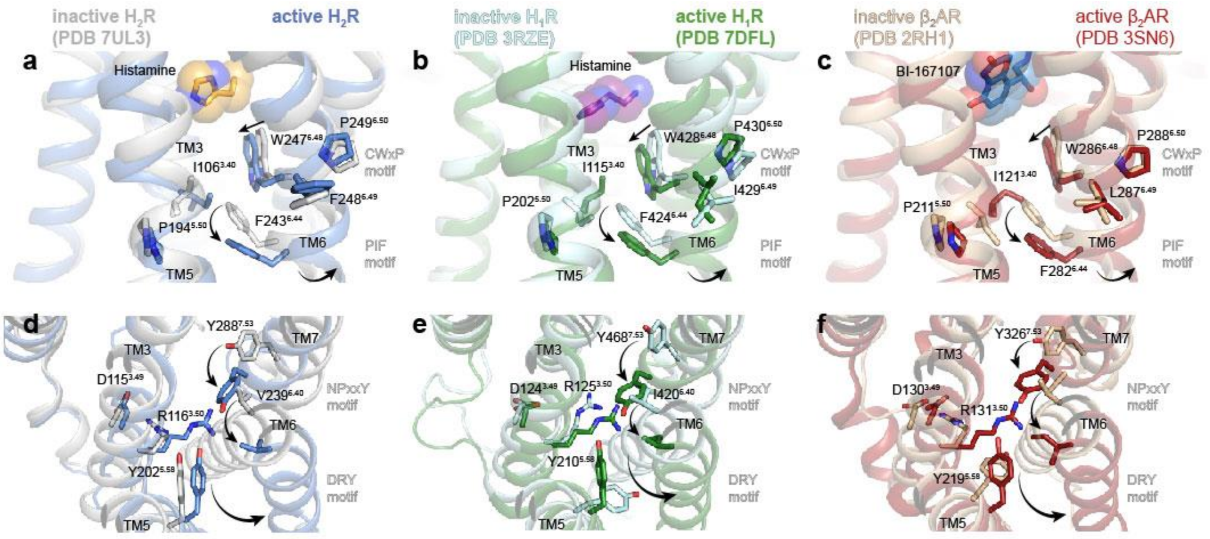

Supplementary Fig. 8. Comparison of the activation mechanism of the H_2R, H_1R and β_2AR. a.-f. Close-up views of the conserved microswitch sequence motifs in the inactive and active structures of the (**a, d**) H_2R, (**b, e**) H_1R, and (**c, f**) β_2AR. The receptors undergo similar conformational changes in the conserved (**a-c**) CWxP and PIF motifs and (**d-f**) NPxxY and DRY motifs, suggesting that these aminergic GPCRs share a similar overall allosteric activation mechanism between the ligand-binding site and the intracellular transducer-coupling cavity. The residues of the conserved microswitches are shown as stick models labeled by residue number and the corresponding Ballesteros-Weinstein numbering (superscript). The receptor-bound agonists are represented as spheres. Arrows indicate the direction of conformational changes upon activation of the receptors.

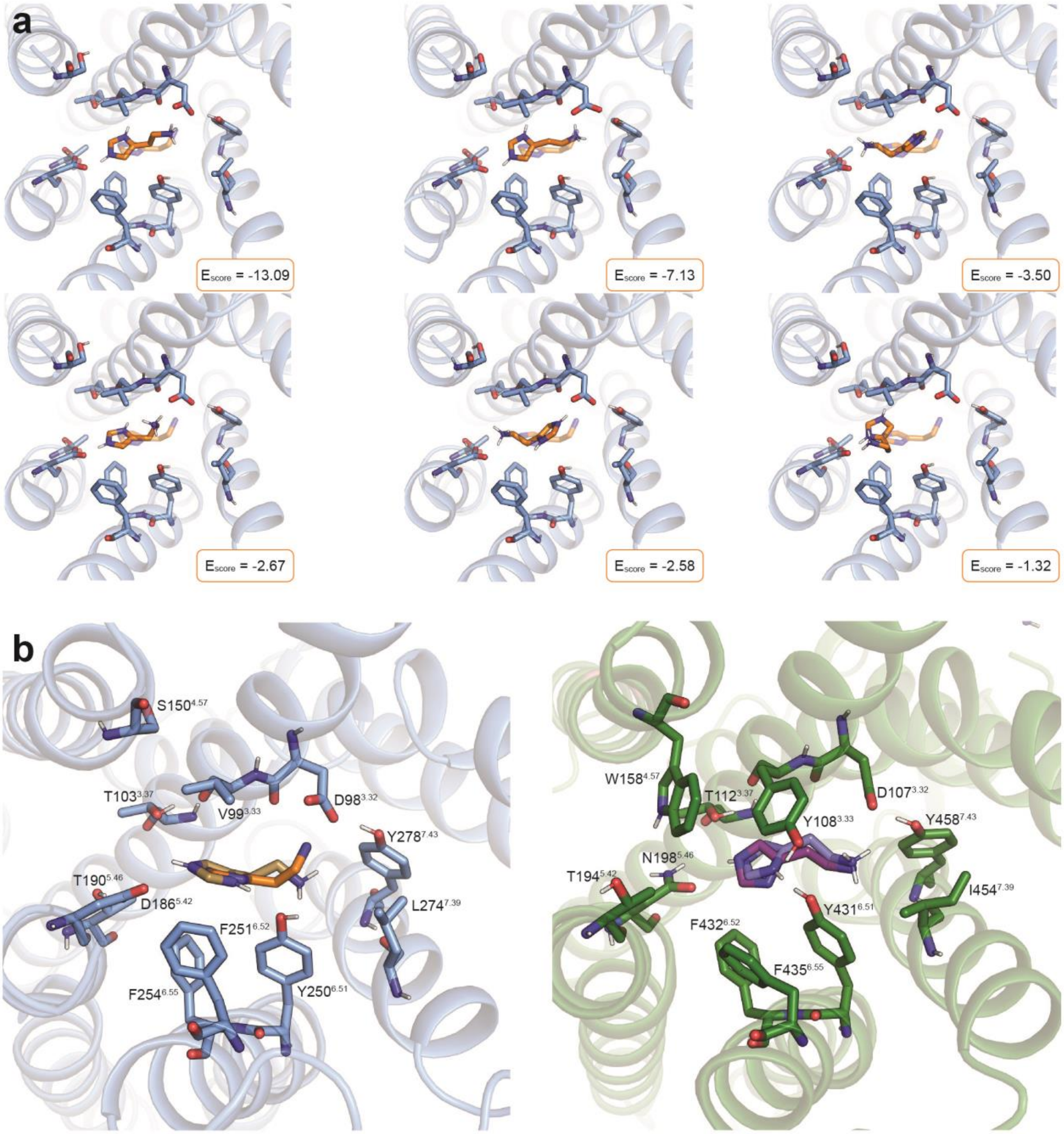

Supplementary Fig. 9. Histamine binding poses. (a) The multiple-copy docking software SEED was used to predict possible alternative binding modes (orange carbons) for histamine in the H_2R in order to assess the likelihood of an alternative orientation compatible with the experimental density. In the orange-framed boxes, the SEED energy score values are reported. The SEED energy score takes into account desolvation and can thus be assumed to yield accurate predictions. Shown are the representatives of the six clusters calculated by SEED in order of descending score. The top-2-scored binding modes are reproducing the experimentally resolved orientation of histamine (yellow carbons), lending additional validity to the experimentally determined orientation. (b) Left: predicted (sand) and experimentally resolved (orange) binding mode of histamine in the H_2R; Right: predicted (violet) and experimentally resolved (red-purple) binding modes of histamine in the H_1R. Binding mode predictions were obtained with AutoDock Vina.

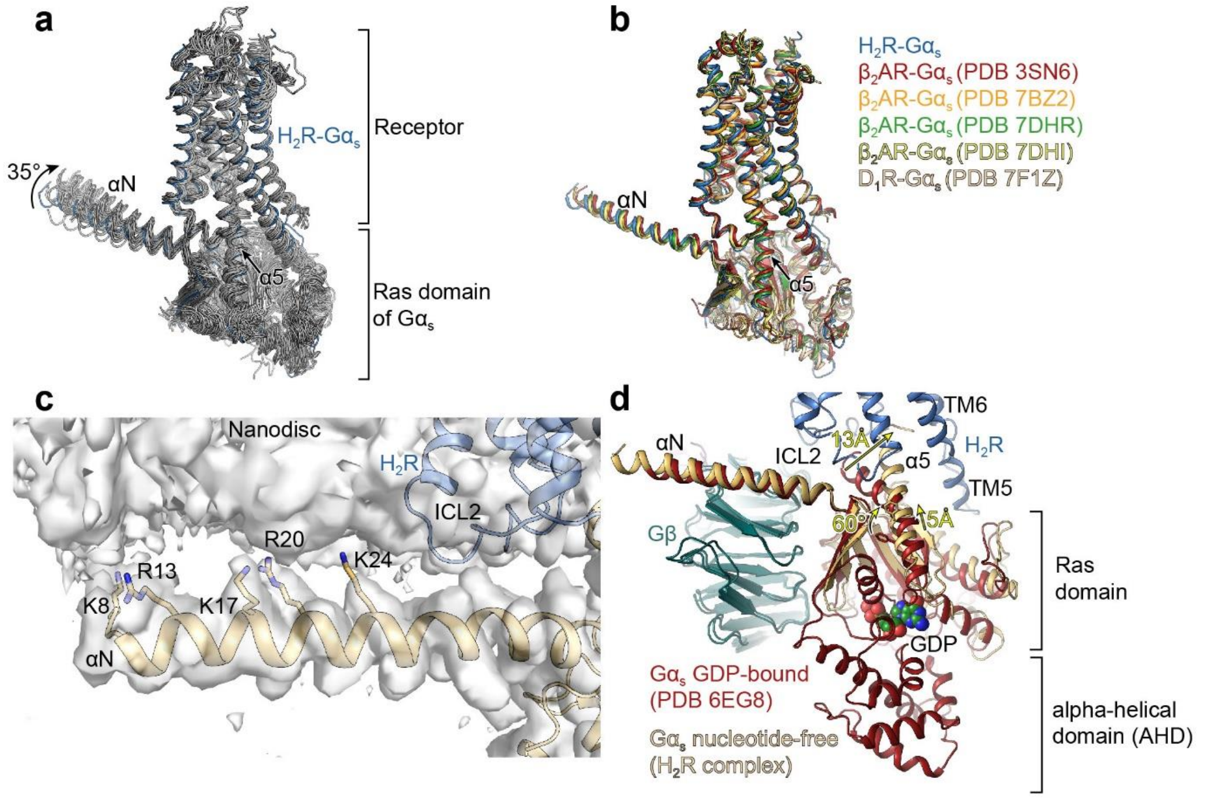

Supplementary Fig. 10. Orientation and interaction of the G protein G_s relative to the receptor and the nanodiscs lipid bilayer. **a.** Comparison of the G protein orientation in aminergic-receptor/G_s complexes (PDB IDs: 7XT9, 7XT8, 7XTB, 7XTC, 8DCR, 8DCS, 7X2F, 7F10, 7X2D, 7F0T, 7F24, 7F1Z, 7F23, 7XJI, 7XJH, 7S0G, 7DH5, 7JOZ, 7CKZ, 7LJC, 7CKW, 7CKY, 7CRH, 7LJD, 7JV5, 7JVQ, 7JVP, 7DHI, 7DHR, 7JJO, 7BZ2, 6NI3, 3SN6) showing differences in the relative orientation of the $G\alpha_s$ subunit with respect to the receptor. The H_2R-G_s complex is highlighted in blue. **b.** Complex structures showing similar receptor-G protein orientations in comparison to the H_2R-$G\alpha_s$ complex. **c.** Interaction of the αN helix of $G\alpha_s$ (wheat) with the nanodisc membrane. Basic lysine and arginine residues that putatively form electrostatic interactions with the polar headgroups of the phospholipids are highlighted as stick models. The H_2R is highlighted in blue. **d.** Structural changes in G_s upon coupling to the H_2R. Major differences between the GDP-bound G_s structure (PDB ID 6EG8) and the nucleotide-free H_2R-$G\alpha_s$ complex involves the rotational translation of the α5 helix into the receptor core and the opening of the alpha-helical domain (AHD) to allow dissociation of the bound GDP (green spheres). Arrows indicate the direction of conformational changes upon activation of the receptors.

Supplementary Table 1. Cryo-EM data collection, refinement, and validation statistics.

	H_2R (EMD- 17793) (PDB 8POK)
**Data collection**	
Microscope	Glacios
Energy filter and camera	Falcon 4 Selectris
Energy filter slit width	10 eV
Voltage (kV)	200
Nominal magnification	130,000
Pixel size (Å)	0.924
Electron exposure (e^{-}/Å^2)	50
Total exposure time (s)	8.17
Number of frames per image	50
Total number of images	46896
Defocus range (µm)	- 0.8 to - 2.0
**Image processing**	
Processing software	cryoSPARC
Motion correction software	cryoSPARC
CTF estimation software	cryoSPARC
Particle selection software	cryoSPARC
Initial particle images (no.)	30.5 M
Final particle images (no.)	425 K
Final refinement software	cryoSPARC
Symmetry imposed	C1
Map resolution (Å)	3.4
B-factor (Å^2)	119.3
**Refinement statistics**	
Initial model (PDB code)	8POK
Modeling software	Coot, PHENIX, model-angelo
Model composition	
Non-hydrogen atoms	8042
Protein residues	1024
Water molecules	0
Ligands	1
Mean *B* factors (Å^2)	
Protein	83.89
Water molecules	-
Ligand	68.59
R.m.s. deviations	
Bond lengths (Å)	0.007
Bond angles (°)	0.978
Validation	
MolProbity score	1.97
Clashscore	7.35
Poor rotamers (%)	0.00

Ramachandran plot	
Favored (%)	89.29
Allowed (%)	10.71
Disallowed (%)	0.00

Supplementary Table 2. AutoDock Vina docking scores for H_2R selective agonists (see Methods section).

VINA	H_1R score	H_2R score
Histamine	-4.8	-4.1
	-4.8	-4.1
	-4.4	-4.0
	-4.0	-4.0
	-3.8	-3.9
	-3.7	-3.9
	-3.7	-3.9
	-3.7	-3.9
	-3.6	-3.9
	-3.6	-3.7
Amthamine	-4.3	-4.9
	-3.9	-4.9
	-3.9	-4.6
	-3.8	-4.5
	-3.8	-4.5
	-3.6	-4.4
	-3.5	-4.4
	-3.5	-4.4
	-3.4	-4.2
	-3.2	-4.2

VINA	H_1R score	H_2R score
Dimaprit	-4.3	-4.6
	-4.1	-4.3
	-4.0	-4.3
	-3.8	-4.1
	-3.8	-4.1
	-3.7	-4.1
	-3.7	-4.1
	-3.7	-4.0
	-3.6	-3.8
	-3.5	-3.6
Bis157	-5.6	-9.0
	-4.8	-8.5
	-4.1	-8.3
	-4.1	-8.2
	-3.9	-7.6
	-3.9	-7.6
	-3.8	-7.5
	-3.8	-7.4
	-3.8	-7.3
	-3.1	-7.1

Supplementary Table 3. Docking scores from different docking softwares for H_1R/H_2R-selective compounds (see Methods section).

	FRED	HYBRID	DOCK
Histamine	-9.6	-6.9	-41.6
Amthamine	-8.2	-6.2	-39.3
Dimaprit	-10.8	-6.8	nd
Bis-157	nd	nd	-31.9
