# Supplementary material for: Cryo-EM structure of cell-free synthesized human histamine 2 receptor/Gs complex in nanodisc environment
Source: Nat Commun. 2024 Feb 28;15:1831. doi: 10.1038/s41467-024-46096-z (PMC10901899; doi:10.1038/s41467-024-46096-z)
Supplement: Supplementary file 3 — Reporting Summary [file 41467_2024_46096_MOESM3_ESM.pdf]

Corresponding author(s): Frank Bernhard
Daniel Hilger
Arne Moeller

Last updated by author(s): Jan 12, 2024

Reporting Summary

Nature Portfolio wishes to improve the reproducibility of the work that we publish. This form provides structure for consistency and transparency in reporting. For further information on Nature Portfolio policies, see our Editorial Policies and the Editorial Policy Checklist.

Statistics

For all statistical analyses, confirm that the following items are present in the figure legend, table legend, main text, or Methods section.

n/a	Confirmed	
☐	☒	The exact sample size (n) for each experimental group/condition, given as a discrete number and unit of measurement
☐	☒	A statement on whether measurements were taken from distinct samples or whether the same sample was measured repeatedly
☐	☒	The statistical test(s) used AND whether they are one- or two-sided<br>*Only common tests should be described solely by name; describe more complex techniques in the Methods section.*
☒	☐	A description of all covariates tested
☒	☐	A description of any assumptions or corrections, such as tests of normality and adjustment for multiple comparisons
☐	☒	A full description of the statistical parameters including central tendency (e.g. means) or other basic estimates (e.g. regression coefficient) AND variation (e.g. standard deviation) or associated estimates of uncertainty (e.g. confidence intervals)
☐	☒	For null hypothesis testing, the test statistic (e.g. F, t, r) with confidence intervals, effect sizes, degrees of freedom and P value noted<br>*Give P values as exact values whenever suitable.*
☒	☐	For Bayesian analysis, information on the choice of priors and Markov chain Monte Carlo settings
☒	☐	For hierarchical and complex designs, identification of the appropriate level for tests and full reporting of outcomes
☒	☐	Estimates of effect sizes (e.g. Cohen's d, Pearson's r), indicating how they were calculated

Our web collection on statistics for biologists contains articles on many of the points above.

Software and code

Policy information about availability of computer code

Data collection	Data were collected on a Glacios 200 kV microscope equipped with a Selectris energy filter. Movies were recorded using a Falcon 4 direct electron detector and EPU software v 2.9.
Data analysis	Data processing was done with cryoSPARC (v.4). Models were computed using ModelAngelo (v.0.3) and refined with Coot (v.0.9) and Phenix (v.1.19). Validation reports were generated by MolProbity. The structure was further analyzed by AlphaFold2 via the COSMIC2 platform using the UCSF Chimera tool MatchMaker (v.1.16). Data are visualized by ChimeraX and Pymol (v. 4.3). Docking was performed by using the Molecular Operating Environment (v.2022.02), the softwares Autodock Vina (v.1.1.2), DOCK3.7 and the OpenEye programs FRED (v.3.3.0.3), HYBRID (v.3.3.0.3), SEED (v.4.0.0) and OMEGA. RDKit software (v. 2018.9.3) was used for RMSD calculations. Data were further analyzed by GraphPad Prism (v.9.5).

For manuscripts utilizing custom algorithms or software that are central to the research but not yet described in published literature, software must be made available to editors and reviewers. We strongly encourage code deposition in a community repository (e.g. GitHub). See the Nature Portfolio guidelines for submitting code & software for further information.

Data

Policy information about availability of data

All manuscripts must include a data availability statement. This statement should provide the following information, where applicable:

- Accession codes, unique identifiers, or web links for publicly available datasets
- A description of any restrictions on data availability
- For clinical datasets or third party data, please ensure that the statement adheres to our policy

The following datasets has been used for structural analysis and comparison: PDB 7DFL [https://doi.org/10.2210/pdb7DFL/pdb], PDB 7UL3 [https://doi.org/10.2210/pdb7UL3/pdb], PDB 3SN6 [https://doi.org/10.2210/pdb3SN6/pdb], PDB 7BZ2 [https://doi.org/10.2210/pdb7BZ2/pdb], PDB 7DHI [https://doi.org/10.2210/pdb7DHI/pdb], PDB 7DHR [https://doi.org/10.2210/pdb7DHR/pdb]), and PDB 7F1Z [https://doi.org/10.2210/pdb7FIZ/pdb].
The EM map for the complete H2R molecule has been deposited in the EMDB under accession code EMD-17793 [https://www.ebi.ac.uk/emdb/EMD-17793].
Atomic coordinates for H2R have been deposited in the Protein Data Bank under the accession code PDB 8POK [https://doi.org/10.2210/pdb8POK/pdb]. Source data are provided with this paper.

Research involving human participants, their data, or biological material

Policy information about studies with human participants or human data. See also policy information about sex, gender (identity/presentation), and sexual orientation and race, ethnicity and racism.

Reporting on sex and gender	does not appl
Reporting on race, ethnicity, or other socially relevant groupings	does not apply
Population characteristics	does not apply
Recruitment	does not apply
Ethics oversight	does not apply

Note that full information on the approval of the study protocol must also be provided in the manuscript.

Field-specific reporting

Please select the one below that is the best fit for your research. If you are not sure, read the appropriate sections before making your selection.

☒ Life sciences ☐ Behavioural & social sciences ☐ Ecological, evolutionary & environmental sciences

For a reference copy of the document with all sections, see nature.com/documents/nr-reporting-summary-flat.pdf

Life sciences study design

All studies must disclose on these points even when the disclosure is negative.

Sample size	Experiments were performed at least in biological triplicates if statistical means had to be calculated.
Data exclusions	no data were excluded
Replication	Experiments were replicated until a clear result was obtained. The exact number of replication is given in the manuscript for each experiment.
Randomization	The study does only cover defined biochemical experiments and does not contain experments with groups of individuals, such as medical filed studies. Randomization is thus not appropriate.
Blinding	The study does only cover defined biochemical experiments and does not contain experments with groups of individuals, such as medical filed studies. Blinding is therefore not appropriate.

Reporting for specific materials, systems and methods

We require information from authors about some types of materials, experimental systems and methods used in many studies. Here, indicate whether each material, system or method listed is relevant to your study. If you are not sure if a list item applies to your research, read the appropriate section before selecting a response.

Materials & experimental systems

n/a	Involved in the study	
	☒	Antibodies
	☒	Eukaryotic cell lines
☒		Palaeontology and archaeology
☒		Animals and other organisms
☒		Clinical data
☒		Dual use research of concern
☒		Plants

Methods

n/a	Involved in the study	
☒		ChIP-seq
☒		Flow cytometry
☒		MRI-based neuroimaging

Antibodies

Antibodies used

Anti-Strep-Tactin-HRP conjugate was obtained from BioRad (catalogue number 1610381)and used in 1:5,000 dilution. Anti-Flag primary antibody and anti-mouse-HRP conjugate were obtained from Sigma (Nrs. F3165 and A9917) and used in 1:1,000 and 1:5,000 dilution, respectively.
Rabbit anti-FLAG M2 antibody (used at 142 ng/mL) and horseradish peroxidase-conjugated goat anti-rabbit antibody (used at 30 ng/ml) were from Cell Signaling Technology, Danvers, MA, USA.
The nanobody35 was produced in the lab by published protocols and used in 15 µM concentration for the formation of H2R/G protein complexes.

Validation

The commercial antibodies are certified by the vendors and links to the analysis certificates are a follows: Anti-Strep-Tactin-HRP conjugate (https://commerce.bio-rad.com/prd/en/US/adirect/biorad?ts=1&cmd=InvoicePDFDisplay&fieldValue=1610380_64490328), anti-Flag primary antibody (https://www.sigmaaldrich.com/certificates/sapfs/PROD/sap/certificate_pdfs/COFA/Q14/F3165-1MG0000280694.pdf), anti-mouse-HRP conjugate (https://www.sigmaaldrich.com/specification-sheets/203/700/A9917-BULK_________SIGMA____.pdf), rabbit anti-FLAG M2 antibody (https://www.cellsignal.com/browse?tab=product&search=Rabbit%20anti-FLAG%20M2%20antibody%20&status=Released,Pending,Released%20On%20Hold,Pre-discontinued) and horseradish peroxidase-conjugated goat anti-rabbit antibody (https://www.cellsignal.com/browse?tab=product&search=horseradish%20peroxidase-conjugated%20goat%20anti-rabbit%20antibody&redirect=true).
In addition, the antibodies are verified in the lab by binding to their cognate epitopes during western blot analysis. The nanobody35 was verified by SDS PAGE analysis and by binding to the G proteins.It was further used to stabilize the GPCR complex and it is clearly visible in the structure. So it was in addition verified by molecular structural analysis.

Eukaryotic cell lines

Policy information about cell lines and Sex and Gender in Research

Cell line source(s)

The HEK293 cells were obtained from Thermofisher Scientific, catalogue number R70507. Trichoplusia ni insect cells were obtained from Expression Systems, catalogue number 94-002F

Authentication

No further authentication of the cell lines was performed.

Mycoplasma contamination

The cell lines were routinely tested using a PCR-based mycoplasma test kit. All cell lines were negative for mycoplasma contamination.

Commonly misidentified lines
(See ICLAC register)

No commonly misidentifed cell lines were used in this study.
